# Physical activity and its associated factors among adults with chronic kidney disease in a community setting: A secondary data analysis

**DOI:** 10.1371/journal.pone.0332604

**Published:** 2025-09-18

**Authors:** Jena Lee, Yujin Suh

**Affiliations:** 1 Lecturer, College of Nursing, JEI University, Incheon, Republic of Korea; 2 The College of Nursing, Healthcare Sciences and the Human Ecology Research Institute, College of Nursing, Healthcare sciences and the Human Ecology, Dong-eui University, Busan, South Korea; University of Luzon, PHILIPPINES

## Abstract

**Background:**

Physical activity in patients with chronic kidney disease is important as it helps lowers blood pressure, reduces inflammatory markers, improves cognitive function, and improves health-related quality of life. However, there is a lack of studies accurately assess physical activity levels in this population.

**Objective:**

This study aimed to examine the characteristics of chronic kidney disease patients across eGFR stages, assess the proportion of Korean adults with chronic kidney disease meeting recommended physical activity levels, and identify the factors associated with meeting the weekly physical activity recommendation.

**Methods:**

We conducted a secondary analysis of data from the 2016–2020 Korean National Health and Nutrition Examination Survey. Descriptive statistics and logistic regression were used to examine physical activity patterns and associated factors.

**Results:**

Among CKD patients, 79.5% engaged in insufficient physical activity. Factors significantly associated with sufficient activity included sex, smoking status, activity limitation, and eGFR.

**Conclusions:**

Tailoring physical activity interventions is crucial, considering the patient’s clinical condition, physical performance, and demographic factors.

## Introduction

Chronic kidney disease (CKD) is a prevalent global health issue, affecting approximately 850 million individuals [1]. CKD significantly contributes to the development of cardiovascular diseases, hypertension, anemia, and bone disorders, while also increasing the risks of mortality [2]. Although many patients with CKD remain in stages 1–4 and do not require dialysis or renal replacement therapy, they often experience reduced physical function and lower levels of physical activity, which are associated with a substantial symptom burden [3].

Reduced physical activity in CKD patients can exacerbate muscle loss [4], increase cardiovascular risk [5], and worsen inflammation and oxidative stress [6]. It may also lead to higher insulin resistance [7] and contribute to poorer mental health [4], further lowering quality of life [5]. Regular physical activity is essential for mitigating these risks and slowing CKD progression.

Despite these challenges, engaging in regular physical activity such as aerobic and resistance activities offers significant benefits for CKD patients [4,7]. Prior research has shown that physical activity provides multiple advantages for patients with CKD Additionally, physical activity has been shown to reduce blood pressure [8,9], improve health-related quality of life (QoL) [10,11], and increase exercise tolerance by boosting maximal oxygen uptake [6,10]. Physical activity may also help reduce certain inflammatory markers [12,13] and has potential cognitive benefits, though more research is needed in this area.

Although the recognized benefits of physical activity, it remains a significant challenge for patients with CKD. A study conducted on 5,656 patients across all CKD stages in England found that physical inactivity worsens as the disease progresses. In CKD Stages 1 and 2, 66% of patients were inactive, and this figure increased significantly to 83% in Stage 3. By Stages 4 and 5, 89% of patients were physically inactive, highlighting the growing prevalence of inactivity as kidney function declines [14]. Similarly, a study encompassing all stages of CKD in South Korea reported that out of 36,732 participants, approximately 42% were classified as physically inactive, based on the criteria of engaging in at least 150 minutes of moderate-intensity or 75 minutes of vigorous-intensity physical activity per week [15].

Several studies have highlighted a range of factors that impact physical activity among individuals with CKD. These factors can be grouped into five primary categories. Demographic factors, such as age [14,16], sex [14], education level [14,17], and employment status [17,18], play a crucial role in influencing physical activity. Physiological factors are also significant, including glomerular filtration rate (GFR) [19,20], hemoglobin (Hb) [14], calcium levels [16], insulin sensitivity [21], body mass index (BMI) [21]. Additionally, physical health factors, including physical functioning, bodily pain, general health [22], as well as the presence of comorbidities [14,17], direct impact patients’ ability to engage in physical activity. Lifestyle factors, particularly smoking [18] and drug use [16], also influence overall physical activity levels in CKD patients. Lastly, psychosocial factors, such as mental health [22], social functioning [22], and self-efficacy [14,18], are critical for understanding the motivations and barriers to physical activity in this population. Although factors influencing physical activity in CKD patients have been identified, there is a lack of studies that accurately assess physical activity levels. Therefore, this study aims to evaluate the physical activity levels in CKD patients and explore associated health outcomes.. Recognizing the impact of physical activity on CKD management is critical for developing effective interventions To address this, we conducted a cross-sectional study among community-dwelling CKD patients in South Korea.

### Aim

The aims of this cross-sectional study were (a) to examine the characteristics of CKD patients at different eGFR stages, (b) examine the proportion of Korean adults with CKD who achieved the recommended level of weekly physical activity, and (c) to identify factors influencing adherence to these recommendations.

## Methods

### Design

A cross-sectional design was adopted to facilitate the survey on physical activity and its associated factors in patients with CKD. This study conducted a secondary analysis using data from the 6th (2016–2018) and partial 7th (2019–2020) Korean National Health and Nutrition Examination Survey. We assessed the 6th (2016–2018) and partial 7th (2019–2020) Korean National Health and Nutrition Examination Survey data for research purpose in 30/09/2024.

### Participants

**Fig 1:**
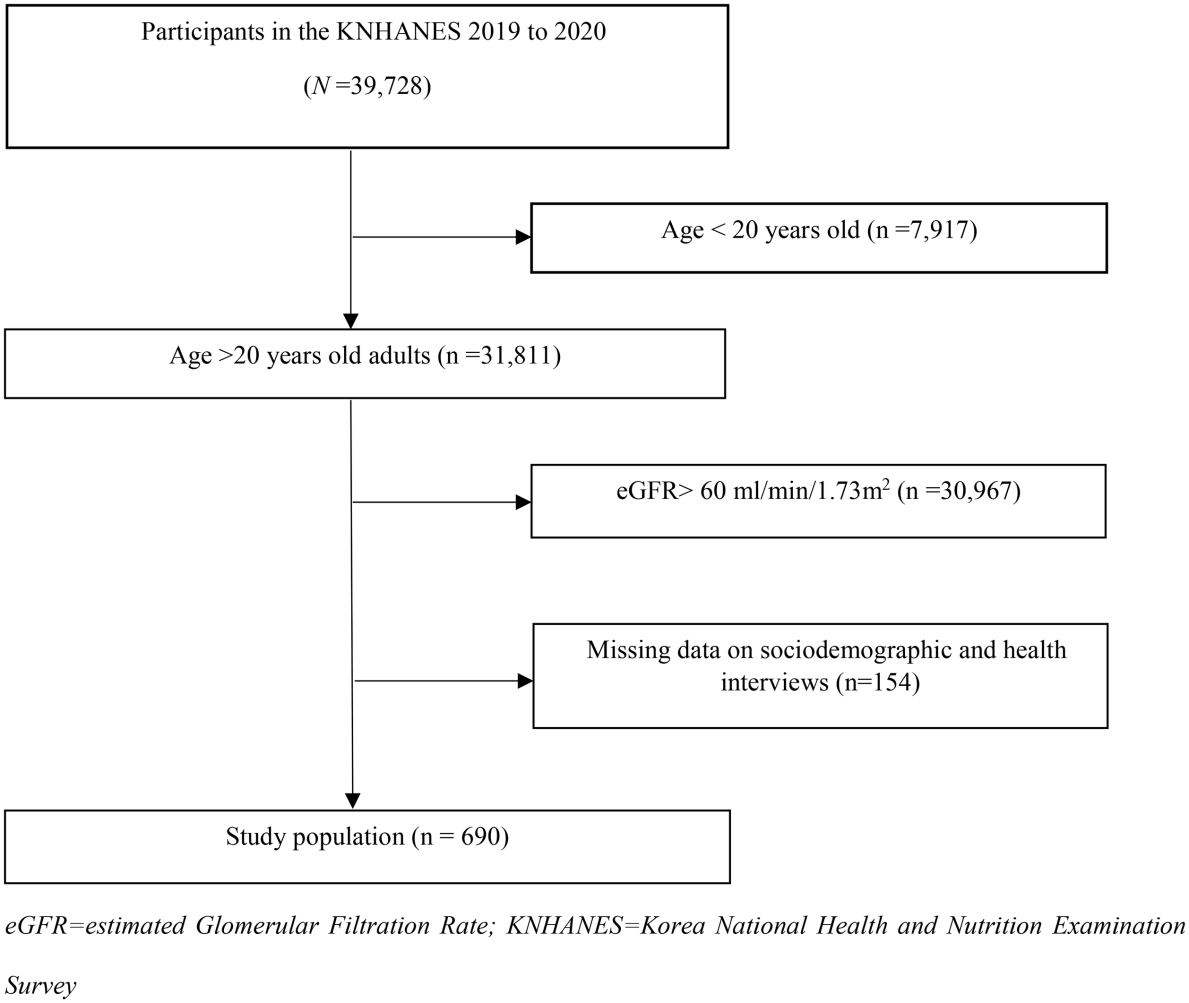
Flow chart for study population. eGFR = estimated Glomerular Filtration Rate; KNHANES = Korea National Health and Nutrition Examination Survey.

This study analyzed data from the 6th and 7th cycles of the Korea National Health and Nutrition Examination Survey (2016–2020). Among 39,728 participants, 31,811 individuals aged 19 years or older were identified, and 844 participants with an eGFR below 60 mL/min/1.73 m² were selected. After excluding cases with missing data, a final sample of 690 participants was included (Fig 1).

### Data collection

The data used in this study were collected from the Korea National Health and Nutrition Examination Survey conducted between January 1, 2016, and December 31, 2020. This included data from the 6th Korean National Health and Nutrition Examination Survey (2016–2018) and the two years of the 7th Korean National Health and Nutrition Examination Survey (2019–2020). The raw data was collected through a health questionnaire, health examination, and nutrition survey. The health interview gathered detailed information on socioeconomic status, health behaviors, quality of life, healthcare utilization, and anthropometric measures. The health examination collected data on biochemical profiles using fasting blood serum and urine; measures of dental health, vision, hearing, and bone density; and X-ray results. The nutrition survey addressed dietary behaviors, food frequency, and food intake. The health interview and health examination were conducted by trained medical staff and interviewers over 3 days for each primary sampling unit at the mobile examination center, which travels to various locations across the country. One week after the health interview, dieticians visit participants at home for the nutrition survey and collect data through face-to-face interviews. Detailed quality control instructions for the survey are available. The Korean National Health and Nutrition Examination Survey data were collected by extracting a representative sample of the entire Korean population using the probability sampling method, and statistical analysis was conducted on the composite sample extraction data.

Data from the 6th (2016–2018) and the first two years of the 7th (2019–2020) KNHANES were combined to increase the sample size and enhance the statistical power of subgroup analyses. According to the guidelines provided from Korea Disease Control and Prevention Agency (KDCA), when integrating multiple survey years, the sampling weights for each cycle are recalculated by dividing the original weights by the number of years in the combined dataset. In this study, the 2-year weights from the 6th cycle and the 2-year weights from the partial 7th cycle were adjusted to represent the Korean adult population over the entire 5-year period (2016–2020). All analyses accounted for the complex, multi-stage sampling design of KNHANES by applying the integrated weights, stratification, and clustering recommended by the KDCA to ensure nationally representative results.

### Measurement

#### Demographic characteristics.

Demographic characteristics included age, sex, education, employment status, and household members. Age was categorized into four groups: 19–39 years, 40–49 years, 50–59 years, and 60 years and older, with data presented as frequencies (N) and percentages (%). Sex was recorded as male or female. Educational attainment was categorized as middle school or less, high school, and college or higher. Employment status was assessed as “yes” for those employed and “no” for those unemployed. Household composition was categorized as living alone, living with one other person, or living with two or more people.

#### Clinical characteristics.

Clinical characteristics included estimated glomerular filtration rate (eGFR), body mass index (BMI), pain status, comorbidities, smoking history, treatment status, depression, self-rated health, and activity limitations. eGFR was calculated using the Chronic Kidney Disease Epidemiology Collaboration (CKD-EPI) creatinine equation [23]. In this study, reduced kidney function was operationally defined based on a single eGFR measurement from cross-sectional data. Participants were categorized into eGFR-based groups: Stage G3 (30–59.9 mL/min/1.73m²), Stage G4 (15–29.9 mL/min/1.73m²), and Stage G5 (<15 mL/min/1.73m²), in alignment with CKD staging guidelines, although chronicity could not be confirmed.

Serum creatinine samples were collected after an 8-hour fast, processed, refrigerated, and transported in cold storage to a central laboratory within 24 hours. BMI was calculated as weight in kilograms divided by height in meters squared, categorized as underweight (<18.5 kg/m²), normal (18.5–24.9 kg/m²), and overweight (≥25.0 kg/m²). Trained investigators measured weight and height using a calibrated scale and stadiometer, respectively, with participants wearing light clothing and no shoes. Pain was assessed by asking participants if they experienced pain, recorded as “yes” or “no.” Comorbidity data were based on self-reported physician-diagnosed conditions and categorized as no comorbidities, one, two, or three or more. Smoking history was divided into nonsmokers, past smokers, and current smokers. Treatment status was classified as “yes” for participants currently receiving treatment and “no” for those not under treatment. Depression was measured through participant responses to the following: “I am not anxious or depressed,” “I am moderately anxious or depressed,” or “I am extremely anxious or depressed.” Responses were categorized as “yes” for moderate or extreme anxiety/depression and “no” for no anxiety/depression. Self-rated health was assessed by the following question, “How do you rate your general health condition?” Participants were asked to rate their health as “very good,” “good,” “fair,” “poor,” or “very poor.” Five ratings for self-rated health were categorized into three groups: very good/ good, fair, and poor/ very poor). Activity limitations were assessed with the following questions: “Are you currently restricted in your daily life and social activities due to problems with your health, either physically or mentally?” The answer was given as “yes” or “no”.

#### Physical activity.

Physical activity was assessed using the Global Physical Activity Questionnaire (GPAQ), developed by the World Health Organization (WHO) and widely used for physical activity evaluation [24]. Participants responded to 15 questions covering three domains: work activity, travel, and recreational activity. For analysis, these were divided into six sub-domains: vigorous work, moderate work, transport, vigorous recreation, and moderate recreation [25]. Total physical activity was calculated by summing the minutes of moderate and vigorous activity, with a conversion factor where 1 minute of vigorous activity equates to 2 minutes of moderate activity [26]. Physical activity was classified as either meeting or not meeting the WHO recommendation of at least 150 minutes of moderate-intensity activity per week [25].

### Ethical considerations

The 6th and partial 7th Korean National Health and Nutrition Examination Survey (2016–2020) were approved by the institutional review board that oversaw the original Korean National Health and Nutrition Examination Survey study. All surveys are conducted with the participants’ written consent to the original Korean National Health and Nutrition Examination Survey protocol. Ethical approval for this secondary data analysis was waived by the Dong-eui University Institutional Review Board (IRB No.: DIRB-202411-HR-W-61), where the analysis for this study was conducted. The research was carried out after obtaining approval for the use of data from Korean National Health and Nutrition Examination Survey by submitting a non-disclosure (confidentiality) agreement and a data use agreement in accordance with the raw data use procedures of the Korean National Health and Nutrition Examination Survey provided by the Korea Disease Prevention and Control Agency.

### Data analysis

This study employed complex sample analysis methods, including stratification, clustering, and weighting, as recommended by the Korean National Health and Nutrition Examination Survey. The statistical procedures used are outlined as follows: First, frequency, mean and standard deviation were reported in the descriptive analysis. Second, the Rao-Scott χ² test was conducted to examine differences in general characteristics according to eGFR and to assess whether adherence to physical activity varied significantly across general characteristics and eGFR levels. Third, logistic regression analysis was performed to identify factors significantly associated with physical activity in the study population.

All statistical analyses were conducted using SPSS Statistics version 26, with a significance level set at p < .05.

## Results

### General characteristics and differences in eGFR according to general characteristics

The mean age of participants was 71.0 years, the majority aged ≥60 years. Slightly more than half were male, and most had an education level of middle school or below, were unemployed, and had a BMI ≥ 25 kg/m². Most participants were non-smokers, reported no activity limitations, and were receiving CKD treatment. eGFR differed significantly by age (*p* < .001), BMI (*p* < .001), and self-rated health (*p* < .001) (Table 1).

**Table 1:** General characteristics of individuals with CKD.

Variables	Categories	Total (N = 690)	Stages			χ2(p)
			G3^1^ (n=625)	G4^2^ (n=47)	G5^3^ (n=18)	
eGFR (mL/min/1.73m2)	M ± SD	47.75 ± 0.53	50.53 ± 0.32	25.13 ± 0.65	9.28 ± 1.04	
Age	19-39	7(1.3)	6(1.2)	0(0.0)	1(8.9)	44.65
	40-49	16(3.4)	13(3.3)	3(5.8)	0(0.0)	**(<.001)**
	50-59	35(7.2)	27(6.0)	3(9.4)	5(39.9)	
	≥60	632(88.1)	579(89.5)	41(84.7)	12(51.2)	
Sex	Male	365(52.9)	336(53.6)	19(38.5)	10(63.3)	4.58
	Female	325(47.1)	289(46.4)	28(61.5)	8(36.7)	(.146)
Education	≤Middle school	464(63.3)	420(63.6)	34(68.4)	10(42.3)	4.97
	High school	133(21.4)	121(21.4)	7(14.7)	5(35.3)	(.489)
	≥College	93(15.4)	84(15.0)	6(16.9)	3(22.4)	
Employment	Yes	196(32.5)	178(32.6)	16(40.3)	2(14.9)	4.01
	No	494(67.5)	447(67.4)	31(59.7)	16(85.1)	(.268)
Household members	1 (Living alone)	170(22.3)	150(21.0)	12(30.0)	8(47.3)	10.38
	2	303(39.8)	280(41.1)	17(31.0)	6(20.2)	(.075)
	≥3	217(37.9)	195(38.0)	18(39.0)	4(32.5)	
BMI(kg/m2)	≥25	305(44.3)	280(45.0)	20(47.3)	5(15.5)	39.10
	18.5 ~ 24.9	372(53.1)	336(53.0)	26(50.8)	10(60.7)	**(<.001)**
	<18.5	13(2.6)	9(2.0)	1(1.8)	3(23.8)	
Pain	Yes	253(35.1)	231(35.7)	18(33.5)	4(18.5)	2.54
	No	437(64.9)	394(64.3)	29(66.5)	14(81.5)	(.297)
Comorbidities	0	46(6.4)	43(6.5)	2(2.0)	1(11.7)	7.68
	1	121(18.5)	110(18.5)	6(14.7)	5(25.3)	(.413)
	2	189(26.6)	176(27.5)	10(17.7)	3(20.3)	
	≥3	334(48.5)	296(47.5)	29(65.5)	9(42.7)	
Smoking	Current	100(16.7)	88(16.4)	9(18.8)	3(23.5)	5.39
	Past	227(31.5)	213(32.3)	9(17.4)	5(35.5)	(.386)
	None	363(51.8)	324(51.3)	29(63.8)	10(40.9)	
Treatment status	Yes	627(91.3)	567(91.0)	43(96.4)	17(88.3)	1.71
	No	63(8.7)	58(9.0)	4(3.6)	1(11.7)	(.486)
Depression	Yes	101(13.1)	92(13.3)	6(11.6)	3(11.1)	0.18
	No	589(86.9)	533(86.7)	41(88.4)	15(88.9)	(.915)
Self-rated health	Good	99(14.2)	93(14.8)	5(10.1)	1(3.2)	27.84
	Fair	296(43.8)	279(46.1)	14(26.1)	3(8.8)	**(<.001)**
	Poor	295(42.1)	253(39.1)	28(63.9)	14(88.0)	
Activity limitations	Yes	149(20.5)	133(20.0)	11(20.9)	5(34.4)	2.42
	No	541(79.5)	492(80.0)	36(79.1)	13(65.6)	(.387)

*BMI = body mass index, eGFR = estimated glomerular filtration rate, M ± SD = mean ± standard deviation,* 1= *eGFR*: 30~59.9, 2= *eGFR*: 15~29.9, 3= *eGFR*: <15.

### The bivariate association of each variable and physical activity in CKD

Physical activity levels in CKD patients varied significantly by sex, education, employment, treatment status, and activity limitations (Table 2).

**Table 2:** The bivariate association of each variable and physical in CKD.

Variables	Categories	Physical activity		χ2	p
		Sufficient^1^	Insufficient^2^		
Total		149(20.5)	541(79.5)		
Age	19-39	3(39.3)	4(60.7)	2.46	.661
	40-49	5(36.0)	11(64.0)		
	50-59	10(22.8)	25(77.2)		
	≥60	156(25.3)	476(74.7)		
Sex	Male	113(31.8)	252(68.2)	15.18	**.001**
	Female	61(18.8)	264(81.2)		
Education	≤Middle school	98(21.3)	366(78.7)	14.08	**.010**
	High school	39(29.7)	94(70.3)		
	≥College	37(38.1)	56(61.9)		
Employment	Yes	63(32.2)	133(67.8)	7.31	**.033**
	No	111(22.6)	383(77.4)		
Household members	1(Living alone)	38(23.5)	132(76.5)	1.24	.623
	2	80(24.8)	223(75.2)		
	≥3	56(28.0)	161(72.0)		
BMI (kg/m2)	≥25	79(27.5)	226(72.5)	1.18	.696
	18.5 ~ 24.9	93(24.4)	279(75.6)		
	<18.5	2(19.8)	11(80.2)		
Pain	Yes	62(24.7)	191(75.3)	0.18	.712
	No	112(26.2)	325(73.8)		
Comorbidities	0	15(37.4)	31(62.6)	3.62	.467
	1	30(24.7)	91(75.3)		
	2	44(23.7)	145(76.3)		
	≥3	85(25.6)	249(74.4)		
Smoking	Current	18(20.1)	82(79.9)	5.69	.152
	Past	74(31.1)	153(68.9)		
	None	82(24.2)	281(75.8)		
Treatment status	Yes	153(24.5)	474(75.5)	5.69	**.038**
	No	21(38.5)	42(61.5)		
Depression	Yes	24(25.0)	77(75.0)	0.03	.879
	No	150(25.8)	439(74.2)		
Self-rated health	Good	27(27.3)	72(72.7)	4.86	.161
	Fair	81(29.2)	215(70.8)		
	Poor	66(21.5)	229(78.5)		
Activity limitations	Yes	26(16.7)	123(83.3)	7.49	**.013**
	No	148(28.0)	393(72.0)		
eGFR (mL/min/1.73m2)	30 ~ 59.9	157(25.6)	468(74.4)	3.24	.332
	15 ~ 29.9	11(20.3)	36(79.7)		
	<15	6(41.5)	12(58.5)		

*BMI = body mass index, eGFR = estimated glomerular filtration rate, M±SD = mean ± standard deviation, 1:* 150-300 min/week, 2: <150 min/week.

Men were more likely to achieve recommended activity levels than women (about 32% vs. 19%), and the proportion increased with education, reaching nearly 38% in those with a college degree. Employed individuals were more active (about 32%) than those not working (23%). Patients receiving treatment and those with activity limitations showed lower adherence (about 25% and 17%, respectively) compared to their counterparts.

### The associated factors of physical activity in CKD

As shown in Table 3, unadjusted analyses indicated that male sex, higher education, employment, absence of treatment, and no activity limitations were associated with a higher likelihood of meeting physical activity recommendations.

**Table 3:** Factors associated with physical activity in individuals with CKD.

Variables	Categories	Unadjusted		Adjusted	
		OR (95% CI)	p	OR (95% CI)	p
Age	≥60	0.52(0.11 ~ 2.52)	.419	1.22(0.27 ~ 5.62)	.796
	50-59	0.45(0.08 ~ 2.56)	.371	0.75(0.14 ~ 3.98)	.738
	40-49	0.87(0.12 ~ 6.16)	.888	1.78(0.27 ~ 11.85)	.551
	19-39	(ref.)		(ref.)	
Sex	Male	2.01(1.34 ~ 3.03)	**.001**	2.56(1.39 ~ 4.70)	**.003**
	Female	(ref.)		(ref.)	
Education	≥College	2.27(1.32 ~ 3.89)	**.003**	1.72(0.91 ~ 3.25)	.095
	High school	1.56(0.93 ~ 2.61)	.094	1.26(0.71 ~ 2.24)	.425
	≤Middle school	(ref.)		(ref.)	
Employment	Yes	1.63(1.04 ~ 2.55)	**.034**	1.37(0.86 ~ 2.19)	.180
	No	(ref.)		(ref.)	
Household members	≥3	1.27(0.75 ~ 2.14)	.375	0.97(0.57 ~ 1.68)	.924
	2	1.07(0.65 ~ 1.78)	.782	0.84(0.49 ~ 1.43)	.515
	1(Living alone)	(ref.)		(ref.)	
BMI(kg/m2)	≥25	1.54(0.30 ~ 7.93)	.605	1.89(0.32 ~ 11.25)	.483
	18.5 ~ 24.9	1.31(0.26 ~ 6.71)	.745	1.51(0.26 ~ 8.94)	.646
	<18.5	(ref.)		(ref.)	
Pain	Yes	0.93(0.62 ~ 1.39)	.712	1.39(0.86 ~ 2.23)	.175
	No	(ref.)		(ref.)	
Comorbidities	≥3	0.58(0.27 ~ 1.23)	.155	2.04(0.46 ~ 8.96)	.344
	2	0.52(0.23 ~ 1.20)	.125	1.57(0.36 ~ 6.96)	.549
	1	0.55(0.23 ~ 1.32)	.180	1.55(0.38 ~ 6.25)	.539
	0	(ref.)		(ref.)	
Smoking	Current	0.79(0.42 ~ 1.51)	.477	0.41(0.18 ~ 0.93)	**.034**
	Past	1.42(0.90 ~ 2.24)	.132	0.75(0.41 ~ 1.38)	.356
	None	(ref.)		(ref.)	
Treatment status	Yes	0.52(0.27 ~ 0.97)	**.040**	0.38(0.11 ~ 1.33)	.131
	No	(ref.)		(ref.)	
Depression	Yes	0.96(0.55 ~ 1.68)	.879	1.15(0.59 ~ 2.21)	.683
	No	(ref.)		(ref.)	
Self-rated health	Good	1.37(0.75 ~ 2.50)	.300	1.02(0.53 ~ 1.98)	.953
	Fair	1.51(0.98 ~ 2.33)	.060	1.35(0.87 ~ 2.10)	.179
	Poor	(ref.)		(ref.)	
Activity limitations	Yes	0.52(0.30 ~ 0.87)	**.014**	0.52(0.28 ~ 0.95)	**.033**
	No	(ref.)		(ref.)	
eGFR(mL/min/1.73m2)	30 ~ 59.9	0.48(0.14 ~ 1.70)	.257	0.26(0.07 ~ 0.98)	**.047**
	15 ~ 29.9	0.36(0.09 ~ 1.50)	.160	0.22(0.05 ~ 1.05)	.057
	<15	(ref.)		(ref.)	

BMI *=* body mass index, eGFR *=* estimated glomerular filtration rate.

After adjusting for sociodemographic and clinical variables, four factors remained statistically significant. Men were more than twice as likely as women to achieve sufficient physical activity (OR = 2.56, 95% CI = 1.39–4.70, *p* = .003). Current smokers had substantially lower odds of being active compared with non-smokers (OR = 0.41, 95% CI = 0.18–0.93, *p* = .034). Participants reporting activity limitations were less likely to meet activity recommendations (OR = 0.52, 95% CI = 0.28–0.95, *p* = .033). Furthermore, individuals with moderate CKD (eGFR 30–59.9 mL/min/1.73m²) had significantly lower odds of sufficient physical activity than those with advanced CKD (<15 mL/min/1.73m²) (OR = 0.26, 95% CI = 0.07–0.98, *p* = .047). Education level, employment, and treatment status were not significant in the adjusted model. The eGFR 15–29.9 (mL/min/1.73m²) group showed a non-significant trend toward lower activity (OR = 0.22, *p* = .057).

## Discussion

This study analyzes the characteristics of individuals with CKD across eGFR stages (Table 1) and finds that most participants were aged 60 or older, with the majority classified in Stage 3. CKD is a chronic condition, with many patients remaining in stages 1–4 without requiring dialysis or renal replacement therapy. Although the number of participants in Stage 5 was small, older adults (≥60 years) represented the majority, emphasizes the prevalence of advanced CKD among the older people. These findings are consistent with previous studies. Wilkinson et al. [14] reported that the median age increased with CKD progression, from 72 years in Stage 3–73 years in Stages 4 and 5, with over 70% of patients aged above 61 in these stages. Similarly, Hara et al. [19] found that the mean age of participants across Stages 3, 4, and 5 was approximately 61 years, with more than 70% being in their 60s across these stages. These results highlight the significant impact of aging on CKD progression.

The distribution of BMI categories differed significantly across CKD stages. A higher BMI (≥25 kg/m²) was prevalent in Stage 3 (45.0%) and Stage 4 (47.3%) but decreased markedly in Stage 5 (15.5%). In contrast, underweight patients (BMI < 18.5 kg/m²) were rare in G3 (2.0%) and G4 (1.8%) but increased significantly in G5 (23.8%). Similarly, Navaneethan et al. [27] found that the number of patients with higher BMI decreases, whereas those with lower BMI increase as CKD stages advance, indicating potential associations with declining renal function and malnutrition.

As CKD progresses, patients tend to rate their health more poorly. A higher proportion of participants in earlier stages (G3) reported good self-rated health, while those in advanced stages (G4 and G5) were more likely to report poor health. This is consistent with findings by Lee et al. [28] and Ko et al. [29], both of which highlighted the link between advanced CKD stages and lower self-rated health. These findings suggest that worsening health perceptions in later CKD stages may reflect functional decline, highlighting the need to address both physical and psychological well-being in CKD care.

In this study, only 20.5% of patients met the recommended physical activity levels, while 79.5% engaged in insufficient activity (Table 2), whereas 44.9% of the general adult population in South Korea were reported to meet the recommended physical activity levels [24]. Consistent with the findings of this study, Chu et al. [20] reported that 78.5% of participants had low physical activity levels. Notably, in their study, only 44.6% of the non- CKD comparison group demonstrated low physical activity levels. Wilkinson et al. [14] reported that 89–94% of participants in CKD stages 4–5 had low physical activity levels, whereas 35–43% of adults in the general population were classified as ‘inactive’. According to previous studies, patients with CKD generally exhibit lower rates of physical activity compared to the general population, highlighting the importance of identifying factors associated with this gap. In addition, male, non-smoking, fewer activity limitations, and lower eGFR were significant factors associated with higher physical activity levels in patients with CKD (Table 3).

Sex is a strong predictor of physical activity in CKD. Men were more likely than women to meet sufficient physical activity criteria. Peng, Han, and Xu [30] also found gender differences in physical activity, with a higher proportion of men being ‘Extremely Highly Active’ at 55.5%, compared to 44.5% in women. Similarly, being female was associated with a higher likelihood of low physical activity, with women being over four times more likely (OR = 4.05) according to Bahadi et al. [16] and nearly twice as likely (OR = 1.668) according to Wilkinson et al. [14]. This difference can be explained by several reasons. Men benefit from elevated testosterone levels, which support cardiovascular health [31]. In contrast, women, particularly those who are middle-aged or postmenopausal, face physiological changes such as declining estrogen levels, impaired autonomic function, and decreased cardiac relaxation, which contribute to difficulties in maintaining regular exercise [30]. Furthermore, societal influences play a significant role, as traditional gender roles encourage men to be more active, while women face cultural barriers that limit their participation [32]. These societal expectations contribute to a higher self-efficacy and greater awareness among men, while women, lacking social support, often experience lower participation [33]. Despite these gender differences, time constraints remain a shared challenge for both men and women, limiting their ability to engage in physical activity [34].

Current smoking status is significantly associated with reduced physical activity levels among patients with CKD in this study. Current smoking reduced the likelihood of physical activity participation by 59% in our study (Table 3), consistent with the 12% reduction reported by Chiang et al. [11]. However, there were few studies on the direct relationship between smoking and low physical activity in patients with CKD. Based on prior research on the health effects of smoking in patients with CKD, it is possible to understand how smoking may influence their physical activity, as follows. Smoking has been shown to induce oxidative stress and inflammation, contributing to impaired muscle function and increased fatigue in patients with CKD [15]. Additionally, the associated decline in pulmonary function and exercise-induced dyspnea further exacerbates limitations in physical activity [35]. Smoking, while used for stress relief, can lead to mental health issues like anxiety and depression, reducing motivation to start and increasing the likelihood of discontinuing physical activity due to physical discomfort [36,37].

The present study found that activity limitation emerged as a key factor influencing physical activity, although it has been infrequently explored in previous research. Variables similar to activity limitation as predictors of physical activity have been used in other studies, including the quality-of-life subdomains ‘physical function’ or ‘role physical’ [17,22,38,39]. Activity limitation in CKD patients is closely associated with chronic musculoskeletal issues, fatigue, and metabolic abnormalities, all of which hinder PA [21,40]. Particularly, dialysis directly exacerbates this limitation by inducing fatigue and muscle weakness, while promoting a sedentary lifestyle due to the extended and repetitive nature of treatment sessions [17,22,39]. The findings suggest that dialysis is a variable that can directly or indirectly influence physical activity levels in patients undergoing hemodialysis.

This study observed an inverse association between eGFR and physical activity, with lower eGFR levels linked to higher activity. This finding contrasts with prior studies that generally reported a positive relationship between kidney function and physical activity [22,41,42]. According to prior research, the influence of eGFR on physical activity is generally explained by the following: higher kidney function is linked to increased physical activity, likely due to reduced inflammation and improved physical capacity [19,43]. In contrast, lower kidney function is associated with greater symptom burden, including fatigue and muscle weakness, which are factors known to reduce physical activity levels [14]. Direct cross-sectional evidence showing higher physical activity among individuals with lower eGFR is limited. However, several observational and programmatic studies indicate that exercise counseling, structured rehabilitation, and group-based interventions can increase activity among patients with advanced CKD. These findings suggest that some patients with lower eGFR in community settings may have increased activity levels in response to clinical advice or participation in local programs [44–46], which may help explain the observed inverse association in our study. Several factors could underlie this unexpected association. Survivor bias may have affected the findings if patients with severe illness and low activity were underrepresented. Self-reported measures could also have introduced misclassification. In addition, patients with lower eGFR levels may have been more likely to adopt healthier behaviors, including physical activity, as part of disease management or in response to clinical advice. Given these considerations, the observed inverse association should be interpreted with caution. Further longitudinal and interventional studies with diverse CKD populations are needed to confirm and better understand this relationship.

Although education, employment, and treatment were significantly associated with physical activity in the unadjusted analysis, they lost statistical significance after adjustment (Table 3), whereas they remained significantly associated with physical activity in the bivariate analysis (Table 2).

Higher education levels were positively associated with physical activity participation in this study, with a higher percentage of patients with college or higher education (38.1%) engaging in physical activity compared to those with lower education levels (21.3%). This is consistent with findings from Peng, Han, and Xu [30]. Lower education levels are often linked to higher physical inactivity, due to limited health awareness and lack of exercise guidance in healthcare settings [16,46].

This study found that the employed (32.2%) were more likely to meet the recommended physical activity levels than the unemployed (22.6%). However, other studies, such as those by Chiang et al. [11] and da Costa Rosa et al. [39], found no significant differences in physical activity based on occupation. While employment alone may not guarantee higher activity levels, job type plays an important role [19]. Sedentary jobs may limit physical activity, while physically demanding jobs may encourage more activity [19,39]. Further research is needed to explore these varying results and the role of different job types in physical activity.

Patients with CKD not receiving treatment were more likely to engage in sufficient physical activity, with 38.5% of them compared to 24.5% of those undergoing treatment. The findings of this study indicate that patients with CKD undergoing treatment encounter multiple barriers to engaging in physical activity, such as demanding therapy schedules, inadequate guidance from healthcare professionals, insufficient exercise counseling, and feelings of powerlessness [18,40]. So, effective communication, information sharing, and the attitudes of healthcare professionals are crucial in promoting physical activity among CKD patients receiving treatment [3,18]. Although treatment status was associated with physical activity in the univariate analysis, this association was no longer significant after adjustment. The use of the self-reported GPAQ may have led to overestimation of physical activity, which should be considered when interpreting the results.

This study revealed that female gender, current smoking, activity limitations, and higher eGFR were associated with lower physical activity in CKD patients. Tailoring physical activity interventions is crucial, considering the patient’s clinical condition, physical performance, and demographic factors [18]. A personalized exercise plan should encourage daily activities, particularly for patients with multiple comorbidities, and adjust treatment schedules and medications to manage fatigue, pain, and emotional distress [40].

## Conclusions

In patients with CKD, female gender, current smoking, activity restrictions, and high eGFR were associated with decreased physical activity. Since reduced physical activity in CKD patients negatively affects quality of life by accelerating muscle loss and increasing cardiovascular risk, individualized physical activity strategies should be implemented based on clinical status, physical function, and demographic characteristics. These findings highlight the need for tailored interventions and counseling in both public health programs and clinical practice to promote physical activity among vulnerable subgroups of the CKD population.

## Limitations

This study has several limitations. First, the small sample size, particularly the limited number of patients with eGFR < 15, may have introduced sampling errors and limit the generalizability of the findings to the broader CKD population. Second, the use of secondary data from the Korean National Health and Nutrition Examination Survey restricted the inclusion of key variables, such as external motivators for physical activity (e.g., medical advice), as well as dialysis status. Because dialysis treatment is known to substantially impact physical activity levels among CKD patients, the absence of this information may have introduced confounding and affected the interpretation of associated factors. In addition, dialysis status was not identified in the dataset, as it is known to significantly influence physical activity in CKD patients and may have affected the interpretation of related factors. Third, physical activity was measured using self-reported data, specifically the Global Physical Activity Questionnaire (GPAQ). Self-reported measures are subject to recall and social desirability biases, which may lead to misclassification of activity levels, thereby influencing the accuracy and validity of the findings. These limitations underscore the need for future research utilizing objective physical activity measurements and inclusion of dialysis status to improve data accuracy and interpretability.

Despite the study’s limitations, this research provides a comprehensive analysis of physical activity among Korean CKD patients using a nationally representative dataset. By examining patients across different eGFR stages, it highlights variations in physical activity and associated factors, addressing gaps in prior research. Furthermore, it identifies modifiable factors, such as smoking and activity limitations, offering practical insights for targeted interventions to improve patient outcomes.

## Supporting information

S1 File STROBE Statement—Checklist of items that should be included in reports of *cross-sectional studies.* (DOCX)

## References

[1] Jager KJ, Kovesdy C, Langham R, Rosenberg M, Jha V, Zoccali C. A single number for advocacy and communication-worldwide more than 850 million individuals have kidney diseases. Nephrol Dial Transplant. 2019;34(11):1803–5. doi: 10.1093/ndt/gfz174 31566230

[2] Levin A, Tonelli M, Bonventre J, Coresh J, Donner J-A, Fogo AB, et al. Global kidney health 2017 and beyond: a roadmap for closing gaps in care, research, and policy. Lancet. 2017;390(10105):1888–917. doi: 10.1016/S0140-6736(17)30788-2 28434650

[3] Baker LA, March DS, Wilkinson TJ, Billany RE, Bishop NC, Castle EM, et al. Clinical practice guideline exercise and lifestyle in chronic kidney disease. BMC Nephrol. 2022;23(1):75. doi: 10.1186/s12882-021-02618-1 35193515 PMC8862368

[4] Perry BG, Mündel T. Lower body positive pressure affects systemic but not cerebral haemodynamics during incremental hyperthermia. Clin Physiol Funct Imaging. 2021;41(2):226–33. doi: 10.1111/cpf.12682 33238075

[5] MacKinnon HJ, Wilkinson TJ, Clarke AL, Gould DW, O’Sullivan TF, Xenophontos S, et al. The association of physical function and physical activity with all-cause mortality and adverse clinical outcomes in nondialysis chronic kidney disease: a systematic review. Ther Adv Chronic Dis. 2018;9(11):209–26. doi: 10.1177/2040622318785575 30364521 PMC6196637

[6] Morishita S, Tsubaki A, Shirai N. Physical function was related to mortality in patients with chronic kidney disease and dialysis. Hemodial Int. 2017;21(4):483–9. doi: 10.1111/hdi.12564 28418625

[7] Thompson S, Wiebe N, Padwal RS, Gyenes G, Headley SAE, Radhakrishnan J, et al. The effect of exercise on blood pressure in chronic kidney disease: A systematic review and meta-analysis of randomized controlled trials. PLoS One. 2019;14(2):e0211032. doi: 10.1371/journal.pone.0211032 30726242 PMC6364898

[8] Barcellos FC, Del Vecchio FB, Reges A, Mielke G, Santos IS, Umpierre D, et al. Exercise in patients with hypertension and chronic kidney disease: a randomized controlled trial. J Hum Hypertens. 2018;32(6):397–407. doi: 10.1038/s41371-018-0055-0 29615792

[9] Wilkinson TJ, Clarke AL, Nixon DGD, Hull KL, Song Y, Burton JO, et al. Prevalence and correlates of physical activity across kidney disease stages: an observational multicentre study. Nephrol Dial Transplant. 2021;36(4):641–9. doi: 10.1093/ndt/gfz235 31725147

[10] Bahadi A, Lagtarna H, Benbria S, Zajjari Y, Elkabbaj D, Zemraoui N. Physical activity in Sahara Moroccan hemodialysis patients. BMC Res Notes. 2021;14(1):65. doi: 10.1186/s13104-021-05460-8 33597008 PMC7890959

[11] Chiang S-L, Lai C-Y, Lee Y-L, Hsu P-H, Hsu Y-J, Chao T-C, et al. Determinants of moderate-to-high physical activity levels in hemodialysis patients: A multicenter cross-sectional study. Nurs Health Sci. 2024;26(3):e13144. doi: 10.1111/nhs.13144 39013554

[12] Van Huffel L, Tomson CRV, Ruige J, Nistor I, Van Biesen W, Bolignano D. Dietary restriction and exercise for diabetic patients with chronic kidney disease: a systematic review. PLoS One. 2014;9(11):e113667. doi: 10.1371/journal.pone.0113667 25423489 PMC4244158

[13] Villanego F, Naranjo J, Vigara LA, Cazorla JM, Montero ME, García T, et al. Impact of physical exercise in patients with chronic kidney disease: Sistematic review and meta-analysis. Nefrologia (Engl Ed). 2020;40(3):237–52. doi: 10.1016/j.nefro.2020.01.002 32305232

[14] Yamagata K, Hoshino J, Sugiyama H, Hanafusa N, Shibagaki Y, Komatsu Y, et al. Clinical practice guideline for renal rehabilitation: systematic reviews and recommendations of exercise therapies in patients with kidney diseases. Ren Replace Ther. 2019;5(1):1–19.

[15] Vanden Wyngaert K, Van Craenenbroeck AH, Van Biesen W, Dhondt A, Tanghe A, Van Ginckel A, et al. The effects of aerobic exercise on eGFR, blood pressure and VO2peak in patients with chronic kidney disease stages 3-4: A systematic review and meta-analysis. PLoS One. 2018;13(9):e0203662. doi: 10.1371/journal.pone.0203662 30204785 PMC6133282

[16] Castaneda C, Gordon PL, Parker RC, Uhlin KL, Roubenoff R, Levey AS. Resistance training to reduce the malnutrition-inflammation complex syndrome of chronic kidney disease. Am J Kidney Dis. 2004;43(4):607–16. doi: 10.1053/j.ajkd.2003.12.025 15042537

[17] Choi S, Jang S-Y, Choi E, Park YS. Association between prevalence and severity of chronic kidney disease and employment status: a nationwide study in Korea. BMC Public Health. 2024;24(1):216. doi: 10.1186/s12889-023-17338-4 38238668 PMC10797861

[18] Hu H, Chau PH, Choi EPH. Physical activity, exercise habits and health-related quality of life in maintenance hemodialysis patients: a multicenter cross-sectional study. J Nephrol. 2024;37(7):1881–91. doi: 10.1007/s40620-024-01935-6 38658480 PMC11519245

[19] Hara M, Nishida Y, Tanaka K, Shimanoe C, Koga K, Furukawa T, et al. Moderate-to-vigorous Physical Activity and Sedentary Behavior Are Independently Associated With Renal Function: A Cross-sectional Study. J Epidemiol. 2023;33(6):285–93. doi: 10.2188/jea.JE20210155 34657911 PMC10165219

[20] Chu NM, Hong J, Harasemiw O, Chen X, Fowler KJ, Dasgupta I, et al. Chronic kidney disease, physical activity and cognitive function in older adults-results from the National Health and Nutrition Examination Survey (2011-2014). Nephrol Dial Transplant. 2022;37(11):2180–9. doi: 10.1093/ndt/gfab338 34850174 PMC9585475

[21] Bowlby W, Zelnick LR, Henry C, Himmelfarb J, Kahn SE, Kestenbaum B, et al. Physical activity and metabolic health in chronic kidney disease: a cross-sectional study. BMC Nephrol. 2016;17(1):187. doi: 10.1186/s12882-016-0400-x 27876008 PMC5120456

[22] Wu Y-H, Hsu Y-J, Tzeng W-C. Physical Activity and Health-Related Quality of Life of Patients on Hemodialysis with Comorbidities: A Cross-Sectional Study. Int J Environ Res Public Health. 2022;19(2):811. doi: 10.3390/ijerph19020811 35055633 PMC8775483

[23] Levey AS, Stevens LA, Schmid CH, Zhang YL, Castro AF 3rd, Feldman HI, et al. A new equation to estimate glomerular filtration rate. Ann Intern Med. 2009;150(9):604–12. doi: 10.7326/0003-4819-150-9-200905050-00006 19414839 PMC2763564

[24] Sung H, Kim G, Ma X, Choe H, Han Y, Yoon J, et al. Physical Activity Trends in Korean Adults from Korea National Health and Nutritional Examination Survey from 2014 to 2019. Int J Environ Res Public Health. 2022;19(9):5213. doi: 10.3390/ijerph19095213 35564610 PMC9100085

[25] Herrmann SD, Heumann KJ, Der Ananian CA, Ainsworth BE. Validity and Reliability of the Global Physical Activity Questionnaire (GPAQ). Measurement in Physical Education and Exercise Science. 2013;17(3):221–35. doi: 10.1080/1091367x.2013.805139

[26] Centers for Disease Control and Prevention. Adult activity: An overview Physical activity basics. 2023 Dec 20. Available from: https://www.cdc.gov/physical-activity-basics/guidelines/adults.html

[27] Navaneethan SD, Schold JD, Arrigain S, Kirwan JP, Nally JV Jr. Body mass index and causes of death in chronic kidney disease. Kidney Int. 2016;89(3):675–82. doi: 10.1016/j.kint.2015.12.002 26880461 PMC4757850

[28] Lee J, Abdel-Kader K, Yabes JG, Cai M, Chang H-H, Jhamb M. Association of Self-Rated Health With Functional Limitations in Patients With CKD. Kidney Med. 2021;3(5):745-752.e1. doi: 10.1016/j.xkme.2021.04.010 34693255 PMC8515078

[29] Ko H-L, Min H-K, Lee S-W. Self-rated health and the risk of incident chronic kidney disease: a community-based Korean study. J Nephrol. 2023;36(3):745–53. doi: 10.1007/s40620-022-01518-3 36477693

[30] Peng W, Han M, Xu G. Gender Differences in the Association between Physical Activity and Mortality in Chronic Kidney Disease: Results from the National Health and Nutrition Examination Survey (2011-2018). J Clin Med. 2023;12(3):779. doi: 10.3390/jcm12030779 36769428 PMC9918191

[31] Gungor O, Ulu S, Hasbal NB, Anker SD, Kalantar-Zadeh K. Effects of hormonal changes on sarcopenia in chronic kidney disease: where are we now and what can we do?. J Cachexia Sarcopenia Muscle. 2021;12(6):1380–92. doi: 10.1002/jcsm.12839 34676694 PMC8718043

[32] Stalling I, Gruber M, Bammann K. Sex differences in physical functioning among older adults: cross-sectional results from the OUTDOOR ACTIVE study. BMC Public Health. 2024;24(1):1766. doi: 10.1186/s12889-024-19218-x 38956507 PMC11221023

[33] Garg S. Gender differences in pathways influencing leisure time physical activity: A structural equation analysis. Diabetes Metab Syndr. 2023;17(5):102761. doi: 10.1016/j.dsx.2023.102761 37119796

[34] García GG, Iyengar A, Kaze F, Kierans C, Padilla-Altamira C, Luyckx VA. Sex and gender differences in chronic kidney disease and access to care around the globe. Semin Nephrol. 2022;42(2):101–13. doi: 10.1016/j.semnephrol.2022.04.001 35718358

[35] Choi HS, Han K-D, Oh TR, Kim CS, Bae EH, Ma SK, et al. Smoking and risk of incident end-stage kidney disease in general population: A Nationwide Population-based Cohort Study from Korea. Sci Rep. 2019;9(1):19511. doi: 10.1038/s41598-019-56113-7 31862942 PMC6925223

[36] Kim M, Kim J, Lee I. Interactive associations of smoking and physical activity with metabolic syndrome in adult men in Korea. Front Public Health. 2023;11:1281530. doi: 10.3389/fpubh.2023.1281530 38035285 PMC10687556

[37] Zhang J, Cao Y, Mo H, Feng R. The association between different types of physical activity and smoking behavior. BMC Psychiatry. 2023;23(1):927. doi: 10.1186/s12888-023-05416-1 38082223 PMC10712079

[38] Qu X, Tong X, Hou X, Zhang J, Hou L, Chen J. Trends in Adherence to Recommended Physical Activity and Its Association with Mortality and Disease Progression among US Adults with Chronic Kidney Disease. Am J Nephrol. 2022;53(8–9):591–602. doi: 10.1159/000526956 36349764 PMC9808653

[39] da Costa Rosa CS, Nishimoto DY, Freitas Júnior IF, Ciolac EG, Monteiro HL. Factors Associated With Levels of Physical Activity in Chronic Kidney Disease Patients Undergoing Hemodialysis: The Role of Dialysis Versus Nondialysis Day. J Phys Act Health. 2017;14(9):726–32. doi: 10.1123/jpah.2016-0715 28513257

[40] Battaglia Y, Baciga F, Bulighin F, Amicone M, Mosconi G, Storari A, et al. Physical activity and exercise in chronic kidney disease: consensus statements from the Physical Exercise Working Group of the Italian Society of Nephrology. J Nephrol. 2024;37(7):1735–65. doi: 10.1007/s40620-024-02049-9 39269600 PMC11519309

[41] Castillo-García A, Valenzuela PL, Saco-Ledo G, Morales JS, Ruilope LM, Santos-Lozano A, et al. Physical activity, chronic kidney disease, and cardiovascular risk: A study in half a million adults. Scand J Med Sci Sports. 2024;34(1):e14557. doi: 10.1111/sms.14557 38268077

[42] Chen A, Wang Y, Wu J, Tang D, Zhu Q, Lu A, et al. Identification and characterization of dynamically regulated hepatitis-related genes in a concanavalin A-induced liver injury model. Aging (Albany NY). 2020;12(22):23187–99. doi: 10.18632/aging.104089 33221747 PMC7746381

[43] Clyne N, Anding-Rost K. Exercise training in chronic kidney disease-effects, expectations and adherence. Clin Kidney J. 2021;14(Suppl 2):ii3–14. doi: 10.1093/ckj/sfab012 33981415 PMC8101627

[44] Anand S, Ziolkowski SL, Bootwala A, Li J, Pham N, Cobb J, et al. Group-Based Exercise in CKD Stage 3b to 4: A Randomized Clinical Trial. Kidney Med. 2021;3(6):951-961.e1. doi: 10.1016/j.xkme.2021.04.022 34939004 PMC8664706

[45] Bohm CJ, Storsley LJ, Hiebert BM, Nelko S, Tangri N, Cheskin LJ, et al. Impact of Exercise Counseling on Physical Function in Chronic Kidney Disease: An Observational Study. Can J Kidney Health Dis. 2018;5:2054358117753615. doi: 10.1177/2054358117753615 29487746 PMC5821295

[46] Tripathy S, Cai X, Adhikari A, Kershaw K, Peralta CA, Kramer H, et al. Association of Educational Attainment With Incidence of CKD in Young Adults. Kidney Int Rep. 2020;5(12):2256–63. doi: 10.1016/j.ekir.2020.09.015 33305119 PMC7710886

